# Cardiac Imaging After Ischemic Stroke or Transient Ischemic Attack

**DOI:** 10.1007/s11910-020-01053-3

**Published:** 2020-06-30

**Authors:** S. Camen, K.G. Haeusler, R.B. Schnabel

**Affiliations:** 1 Clinic for Cardiology, University Heart and Vascular Center Hamburg, Hamburg, Germany; 2 DZHK (German Center for Cardiovascular Research) (partner site Hamburg/Kiel/Luebeck), Berlin, Germany; 3 Department of Neurology, Universitätsklinikum Würzburg, Würzburg, Germany; 4 University Heart Center Hamburg-Eppendorf, Martinistrasse 52, 20246 Hamburg, Germany

**Keywords:** Ischemic stroke, Transient ischemic attack, Echocardiography, Magnetic resonance imaging, Computed tomography, Embolism

## Abstract

**Purpose of Review:**

Cardiac imaging after ischemic stroke or transient ischemic attack (TIA) is used to identify potential sources of cardioembolism, to classify stroke etiology leading to changes in secondary stroke prevention, and to detect frequent comorbidities. This article summarizes the latest research on this topic and provides an approach to clinical practice to use cardiac imaging after stroke.

**Recent Findings:**

Echocardiography remains the primary imaging method for cardiac work-up after stroke. Recent echocardiography studies further demonstrated promising results regarding the prediction of non-permanent atrial fibrillation after ischemic stroke. Cardiac magnetic resonance imaging and computed tomography have been tested for their diagnostic value, in particular in patients with cryptogenic stroke, and can be considered as second line methods, providing complementary information in selected stroke patients.

**Summary:**

Cardiac imaging after ischemic stroke or TIA reveals a potential causal condition in a subset of patients. Whether systematic application of cardiac imaging improves outcome after stroke remains to be established.

## Introduction

**Fig. 1:**
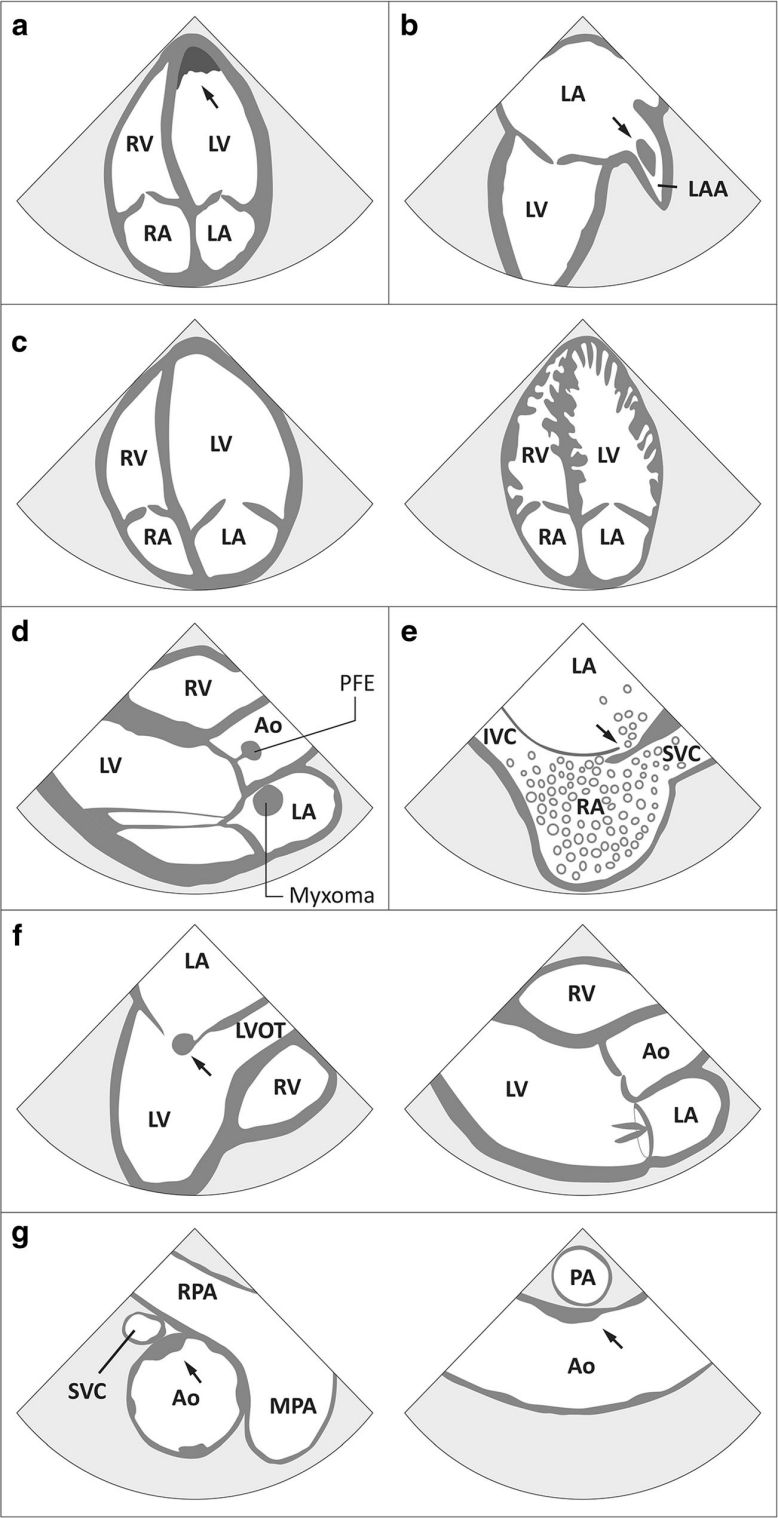
Schematic presentation of the most frequent sources for cardiovascular embolism. **a** The most common localization of *LV thrombi*, usually the result of regional akinesia due to a previous myocardial infarction, is the LV apex, which is ideally visualized on the apical 4-chamber view of the TTE. **b** Typical presentation of a *LAA thrombus* on a TEE mid-esophageal 2-chamber view at about 90°. **c** Transthoracic apical 4-chamber-view demonstrating the pronounced dilation of the left ventricle in case of a *dilated cardiomyopathy* (left) and the multiple prominent ventricular trabeculations with intertrabecular spaces seen in *non-compaction cardiomyopathy* (right). **d** The usually valve associated *papillary fibroelastoma (PFE)* and *myxoma*, typically located in the atria, are the most common primary cardiac tumors in adults, which are both associated with a high risk of embolism (TTE parasternal long-axis view). **e** Bubble transition from the right to the left atrium (positive “bubble test”) in case of a *patent foramen ovale* documented using a TEE mid-esophageal bicaval view at about 110°. **f** Vegetations, a main criterion for *endocarditis* (here mitral valve endocarditis diagnosed in a mid-esophageal longitudinal axis view of the left ventricle at about 120° in the TEE examination; left), and *prosthetic valves* (here double-wing prosthesis of the mitral valve shown in a parasternal longitudinal axis view of the TTE; right) are further potential sources of cardioembolism. **g***Aortic atheroma* ≥ 4 mm have been associated with ischemic stroke and can be detected during retraction of the TEE probe at the end of the examination (here mid-esophageal short (left) and long (right) axis view of the ascending aorta). Ao aorta, IVC inferior vena cava, LA left atrium, LAA left atrial appendage, LV left ventricle, LVOT left ventricular outflow tract, (M/R) PA (main/right) pulmonary artery, RA right atrium, RV right ventricle

Ischemic stroke etiology can be divided into five categories based on the TOAST criteria [1]: large artery atherosclerosis, cardioembolism, small vessel occlusion, stroke of other determined etiology, and stroke of undetermined (“cryptogenic”) etiology. Cardioembolic stroke accounts for about 20–25% of all ischemic strokes [2]. Sources for cardioembolism are further classified into major- or minor-risk sources according to their (thrombo-) embolic potential [3]. The most common major-risk source of cardioembolism is atrial fibrillation (AF) [4]. Less frequent major-risk sources are cardiomyopathies with left ventricular (LV) dysfunction, intracardiac thrombi, cardiac tumors, prosthetic valves, and endocarditis [3] (Fig. 1). Minor-risk sources include patent foramen ovale (PFO), atrial septum aneurysm (ASA), and calcification of aortic and mitral valve [3].

Transthoracic (TTE) and/or transesophageal echocardiography (TEE) are the standard methods for cardiac imaging after ischemic stroke [5, 6]. In recent years, reports on the use of cardiac magnetic resonance imaging (MRI) and computed tomography (CT) after stroke have been increasingly published, particularly in patients with unknown stroke etiology [7–10]. Cardiac work-up after stroke might lead to changes in secondary stroke prevention, guide screening for atrial fibrillation, and serve as a screening tool for (coronary) heart disease given the strong overlap of cerebrovascular and cardiovascular risk factors [11, 12]. While screening for AF after stroke has become common practice, cardiac imaging after ischemic stroke is less well established [4, 11, 13].

The purpose of this review is to summarize the available evidence, latest findings and guideline recommendations to guide physicians in the use of cardiac imaging after ischemic stroke or transient ischemic attack (TIA).

## Cardiac Imaging and Stroke Etiology

The primary goal of cardiac imaging after stroke is to identify potential sources of embolism and subsequently establish stroke etiology. Due to the lack of prospective, randomized trials, the guideline recommendations for cardiac imaging after stroke differ considerably, resulting in a highly varying use of TTE and TEE across stroke centers [14]. Furthermore, the two most recent prospective studies comparing TTE and TEE in ischemic stroke patients were conducted in 2006 and yielded inconclusive results. In a prospective single-center study including 503 ischemic stroke patients, the incremental value of TEE over routine work-up (including TTE, carotid ultrasound, 12-lead electrocardiogram (ECG) and 24-h Holter-ECG in individuals with high suspicion for AF) was investigated [15]. Based on TEE findings, OAC was prescribed in 8% of 212 patients with so far cryptogenic ischemic stroke. However, in the vast majority of cases the indication for oral anticoagulation was at least debatable (e.g., aortic thrombi). Another prospective single-center TEE study included patients with an ischemic stroke or a TIA and no indication for OAC after routine work-up (including TTE, carotid ultrasound, ECG, and blood test) [16]. Besides an increased detection of aortic plaques and interatrial septum abnormalities using TEE, cardiac thrombi, almost exclusively located in the LA appendage (LAA), were found in 17% of the 231 patients compared to 1% of the patients who were examined with TTE only. However, no prolonged rhythm monitoring was performed and the incidence of newly detected AF was not reported. Considering that the detection of LAA thrombi in individuals with sinus rhythm and normal TTE is a rare finding [17, 18], it seems likely that there is a high rate of so far undetected AF in individuals with LAA thrombus. In a small retrospective single-center study, additional findings using TEE were again mainly related to interatrial septum abnormalities [19]. A retrospective analysis of the prospective FindAF RANDOMISED trial—including ischemic stroke patients aged 60 years and older—revealed that TEE findings led to a change in therapy in 14 out of 89 selected patients, whereas TTE findings led to a change in therapy in 1 out of 90 selected patients [20]. However, only a minority of patients received both TTE and TEE and the decision for TTE or TEE was not based on predefined criteria, limiting the direct comparison of both examinations. Furthermore, a significant proportion of the therapy changes based on TEE findings is debatable (mainly OAC in case of a newly diagnosed PFO). In a prospective multi-center observational study including 603 TIA patients, echocardiography (mainly TTE) revealed a potential source of cardioembolism in 10%, with a higher yield in patients with known coronary artery disease (CAD) or in the presence of acute infarction on brain MRI. However, changes in medical therapy based on echocardiography were rare, which led the authors to conclude that the decision to perform echocardiography in TIA patients should be individualized [21].

Since stroke etiology remains unexplained in a relevant proportion of individuals after standard work-up including echocardiography, the incremental value of cardiac MRI and CT has been investigated [22]. In a prospective single-center study, 103 patients with cryptogenic stroke according to routine work-up including TEE and in most cases TTE were assigned to receive an additional MRI examination of the heart and the aorta [7••]. Although stroke classification based on findings from both imaging modalities was consistent in the vast majority of cases (86%), cardiac MRI identified a potential source of embolism in additional 6.1%, including four patients with regional wall motion abnormalities izn ≥ 3 segments and one patient with aortic plaques ≥ 4 mm. However, MRI missed echocardiography-detected sources of embolism in seven patients. In five of these seven cases, MRI had to be terminated prematurely due to intolerance by the patients or the need for an emergency examination in a different patient. The remaining two had a PFO combined with an ASA. In an earlier prospective single-center study, the additional benefit of cardiac MRI over routine work-up including TTE, but not TEE, was evaluated in 85 ischemic stroke patients [8]. Cardiac MRI identified a potential source of embolism in 6 (26%) of the 23 patients who had been classified as a cryptogenic stroke after routine work-up. Three stroke patients had an evident embolic source (two LV thrombi and one complex aortic thrombus), whereas the other three were interatrial septum abnormalities (two ASAs, one ASA + PFO). The authors did not report on any pathological finding detected on TTE.

To date, the largest prospective single-center study comparing the combined CT examination of the heart, aorta and brain-supplying arteries with TTE and TEE enrolled 140 patients with the suspicion of cardioembolic stroke or TIA mainly based on brain imaging findings [9•]. Patients with AF on the admission ECG were excluded. Electrocardiogram-gated CT examinations revealed prior myocardial infarction (MI) more accurately than echocardiography and also detected an LV-thrombus in one additional patient, whereas three small thrombi in the LAA were missed by cardiac CT. The authors concluded that cardiac CT might offer complementary information for stroke classification. Another prospective single-center study compared cardiac CT with TTE and TEE in 46 unselected ischemic stroke patients [23]. With TEE as reference, cardiac CT had a sensitivity of 72% for the identification of potential cardiac source of embolism, with one LV-thrombus only detected by cardiac CT. In an earlier single-center prospective study including 137 patients with suspicion for a cardioembolic stroke and a high cardiovascular risk profile, cardiac CT yielded similar results compared to TEE regarding high-risk sources of embolism [24]. However, many low-risk sources of embolism such as PFO/ASA were missed using cardiac CT only. In a small prospective single-center pilot study of 20 ischemic stroke patients, the feasibility of simultaneous cardiac CT examination along with the initial brain imaging was tested [25]. Cardiac CT revealed one LV-thrombus and one LAA-thrombus, both validated using TEE the next day. Furthermore, one patient was diagnosed with a Stanford type A aortic dissection.

The advantages, disadvantages, and a comparison of the diagnostic value of all four cardiac imaging modalities are summarized in Table 1.

**Table 1:** Advantages, disadvantages, and diagnostic value of cardiac imaging methods (modified according to [10])

	TTE	TEE	Cardiac MRI	Cardiac CT
Advantages	• Readily available • Cheap • Non-invasive	• Excellent spatial and temporal resolution	• Good spatial resolution • Excellent tissue characterization	• Excellent spatial resolution • Fast acquisition
Disadvantages	• Operator dependent • Limited by patient characteristics (e.g., obesity, lung disease)	• Operator dependent • Semi-invasive • Usually requires sedation	• Gadolinium exposure • Most expensive • Requires ability to hold breath • May require sedation • Limited by patient characteristics (e.g., pacemaker)	• Radiation • Iodinated contrast agent exposure • Expensive
Cardiomyopathy	++	+	+++*	–
LV-thrombus	++	+	+++*	++
PFO	++	+++*	–	+
Valvular disease	++	+++*	–	++ (+++)*^, #^
Cardiac tumors	+	++	+++*	++
LA/LAA	+	+++*	++	++
Aortic atheroma	–	++	++	+++*

*CT* computed tomography, *LA* left atrium, *LAA* left atrial appendage, *LV* left ventricle, *MRI* magnetic resonance imaging, *PFO*, patent foramen ovale, *TEE* transesophageal echocardiography, *TTE* transthoracic echocardiography

*Diagnostic gold standard

^#^Offers supplemental information to TEE with regard to paravalvular extent of the disease and in patients with prosthetic valves

## Cardiac Imaging for Sources of Stroke

### (Ischemic) Cardiomyopathy and Left Ventricular Thrombus

Coronary artery disease is a common finding in patients with ischemic stroke. Coronary CT studies revealed obstructive CAD in up to 25% of symptom-free ischemic stroke patients without previously known CAD [26]. However, screening for occult CAD in stroke patients remains a matter of debate and current guidelines do not provide clear recommendations [12, 27]. Further diagnostic approaches in the acute phase after stroke might be guided by high-sensitivity assayed troponin concentrations, routinely used in the diagnosis of acute MI, which have been shown to be elevated frequently in ischemic stroke patients [28]. However, in a recent prospective two-center study, only 7 of 29 ischemic stroke patients with elevated troponin concentrations who were referred to coronary angiography were diagnosed with a coronary culprit lesion, whereas 14 patients had no obstructive CAD at all [29]. Results from a small sub-study of nine patients suggest that cardiac MRI might be helpful to select stroke patients in need for an urgent coronary angiography, since it enables the detection of late gadolinium enhancement—consistent with prior MI—and subtle wall motion abnormalities not detected on TTE [30]. In a recent study, late gadolinium enhancement was detected in 15% of stroke patients without known CAD indicating the need for further cardiac diagnostic work-up (e.g., non-invasive myocardial stress tests) [7••]. Signs of MI in stroke patients undetected on TTE have also been demonstrated using cardiac CT [9•].

Regional wall motion abnormalities due to prior MI are the most common reason for the formation of LV-thrombi, although the incidence has significantly declined due to improved reperfusion strategies. However, LV-thrombi may still occur in up to 8% of patients after ST-elevation MI [31, 32]. According to a meta-analysis, LV-thrombi are associated with an increased risk for ischemic stroke that can be reduced by taking vitamin K antagonist OAC [33]. Cardiac MRI possesses higher sensitivity and specificity compared to TTE/TEE, rendering it the gold standard for the detection of LV-thrombi [34, 35]. Since many LV-thrombi are located in apical region of the LV, TTE is most frequently used for the detection of LV-thrombi and may serve as an initial screening test (Fig. 1a) [31, 34]. The diagnostic yield of TTE can be improved by applying a contrast agent [35]. However, if suspicion for a LV-thrombus is high despite negative findings on TTE, a cardiac MRI should be performed [36•]. In a recent prospective single-center study including 60 ischemic stroke patients with an LV ejection fraction < 50% or a previous MI, cardiac MRI revealed LV-thrombi in twelve patients compared to one patient using TTE [36•]. The detection of an LV-thrombus was associated with a reduced LV ejection fraction compared to individuals without LV-thrombus. If cardiac MRI is not easily available, a CT might be considered as an alternative [9, 23].

Apart from LV-thrombi, other cardiomyopathies such as dilated or the rare LV non-compaction cardiomyopathy have also been linked to ischemic stroke [37–39]. While both diseases lead to a significant reduction of the LV ejection fraction, dilated cardiomyopathy is characterized by dilatation of the LV, whereas multiple prominent ventricular trabeculations with intertrabecular spaces can be found in non-compaction cardiomyopathy [37, 38]. Initial screening can be performed by TTE, preferably using contrast agent in case of suspicion for non-compaction in order to unmask the deep intertrabecular recesses (Fig. 1c) [3, 38]. Once more, cardiac MRI can secure the diagnosis and exclude LV thrombi.

### Patent Foramen Ovale

While a PFO can be found in about 25% of the general population, its prevalence in young patients with cryptogenic stroke is reported to be up to 54% [40, 41]. It represents a potential mechanism for paradoxical embolism via a right-to-left shunt and is associated with an increased risk of stroke recurrence in the presence of an ASA [42, 43]. In 2017 and 2018, three randomized open-label trials were able to demonstrate a significant reduction of stroke recurrence rate by interventional PFO-closure compared to antiplatelets in patients with cryptogenic stroke aged 18–60 years [44, 45]. Based on the currently available data, an interventional PFO-closure should be considered in selected patients with cryptogenic stroke, aged ≤ 60 years with moderate to high atrial shunt volume after prior rigorous work-up including at least 72 h of rhythm monitoring.

A first screening for a PFO can be conducted using TTE with an intravenous injection of an agitated air-saline solution. A bubble transition from the right to the left atrium indicates an interatrial septum abnormality (“positive bubble test”). This procedure should be repeated while the patient performs a Valsalva maneuver, which has been shown to significantly improve PFO detection [46]. Large right-to-left shunts can usually be detected using TTE, while smaller shunts are frequently missed [47]. Transcranial Doppler examination after intravenous injection of agitated air-saline solution, gelatin-based solutions or Echovist® (Schering, Berlin, Germany) is a reliable alternative for initial screening for atrial right-to-left shunting in stroke patients. According to a meta-analysis of prospective studies with TEE as the reference, the weighted mean sensitivity and specificity of transcranial Doppler examination was 97% and 93%, respectively [48]. In order to establish the exact anatomy and the precise shunt volume, a TEE examination including the bubble-test is necessary and is considered the gold standard for PFO diagnosis (Fig. 1e) [47]. However, there are case reports indicating bubble transition to the left atrium due to a pulmonary arteriovenous malformation in the absence of an atrial shunt [49].

Cardiac CT showed inferior results compared to TEE with regard to the detection of interatrial septum abnormalities [23, 24, 50]. In a retrospective single-center study including 152 stroke patients, a shunt was found in 19 out of 26 patients with TEE-detected PFO with two false positive results, resulting in a sensitivity and specificity of 73% and 98%, respectively [50]. Nevertheless, cardiac CT might be an alternative in patients with contraindications for TEE, whereas cardiac MRI is not well suited for the diagnosis of a PFO [7, 51, 52].

### Valvular Disease

Septic cerebral embolism occurs in up to 64% of patients with left-sided infective endocarditis with many episodes being silent and only detectable on cranial MRI [53]. European guidelines recommend TTE for initial screening in case of suspected infective endocarditis [54]. However, TEE is superior to TTE in diagnosing infective endocarditis, in particular in case of small vegetations, poor image quality or prosthetic heart valves (Fig. 1f) [55]. If clinical suspicion persists despite a negative TTE, a TEE examination should be performed and repeated if considered necessary [54]. Patients with a positive TTE should always undergo TEE for further assessment [54]. Echocardiography findings can aid risk stratification and guide therapeutic management based on vegetation size and degree of valvular destruction/insufficiency [54]. Cardiac CT is a valuable alternative to TEE, in particular in patients with prosthetic valve infective endocarditis, in whom acoustic shadowing might impair visualization of the valves [56, 57]. While small vegetations and leaflet perforations may be missed, CT is often superior to TEE for the assessment of the perivalvular extent of the disease [56, 57]. The combination of positron emission tomography with CT can be helpful in case of inconclusive results [54].

Another potential source of cardioembolic stroke in patients with prosthetic valves is thrombus formation, often caused by inadequate anticoagulation [58]. Valve thrombosis is more common in mitral valve replacement than in aortic valve replacement and in mechanical prostheses compared to bioprostheses [59, 60]. Possible dysfunction of the valves might be revealed on TTE, but TEE should be performed for validation of the diagnosis [61]. In case of inconclusive results after TEE, cardiac CT can be performed for further clarification [61].

### Cardiac Tumors

Cardiac tumors are rare sources of cardioembolism. The most common primary cardiac tumors in adults are myxomas, usually found in the LA, and the valve-associated papillary fibroelastomas (Fig. 1d) [3, 62, 63]. Both are benign and often first diagnosed after cardioembolic stroke [64, 65]. Initial screening can be achieved by echocardiography with TEE providing higher sensitivity and spatial resolution [66]. Cardiac CT and in particular MRI can be performed additionally to obtain information on the exact extent of the tumor, its vascularization and tissue characterization and to facilitate differential diagnosis [63].

### Left Atrial (Appendage) Thrombus

The LA and, in particular, the LAA are the typical location for thrombus formation in patients with AF (Fig. 1b) [67]. TEE is considered the gold standard for LA/LAA thrombus evaluation [16]. Nevertheless, cardiac CT might be a valuable alternative as it is non-invasive and offers a superior visualization of the LAA anatomy compared to TEE [68]. In a meta-analysis of 19 studies, the mean sensitivity and specificity of cardiac CT was 96% and 92% compared to TEE, respectively [69]. Focusing on seven mostly prospective, single-center studies in which delayed imaging CT was performed further increased sensitivity (100%) and specificity (99%). Cardiac MRI has also been shown to be a reliable alternative for the evaluation of the LAA [70]. In a retrospective analysis of 261 registry patients undergoing pulmonary vein isolation, delayed enhancement cardiac MRI detected all nine thrombi found on TEE [71]. In line with this, an earlier prospective single-center study including 50 patients with prior cardioembolic stroke and persistent non-valvular AF revealed that cardiac MRI detected all TEE-detected thrombi, but three patients had false positive MRI findings due to artifacts [72]. Since the pathophysiological relationship between the presence of LA/LAA thrombi and cardioembolic stroke appears obvious, imaging of the LA/LAA in patients with stroke (of unknown etiology) seems to be a reasonable approach. However, LA/LAA thrombi are rarely found in patients with continuous sinus rhythm and without specific structural cardiac pathologies [17, 18].

## Cardiac Imaging for Atrial Cardiomyopathy and Prediction of Poststroke Atrial Fibrillation

Of note, the prevalence of cryptogenic stroke according to TOAST criteria is about 25–30%, including patients with (in) complete diagnostic evaluation and competing stroke etiologies [2, 4, 22]. Since the diagnostic work-up for cryptogenic stroke was not established, the term “embolic stroke of undetermined source (ESUS)” has been introduced [73]. ESUS is defined as an imaging-confirmed non-lacunar stroke without extra- or intracranial atherosclerosis causing ≥ 50% stenosis in an artery supplying the area of recent brain ischemia, apparent major-risk cardioembolic source determined by echocardiography (using TTE or TEE), no episode of AF ≥ 6 min within 24 h of ECG monitoring, and no other specific cause of stroke [73]. Results from long-term ECG monitoring led to the assumption, that a significant proportion of cryptogenic stroke as well as ESUS might have been caused by subclinical AF as well [4, 11]. As a clear biological gradient between AF burden and stroke risk has to be questioned, the concept of atrial cardiomyopathy as an alternative source of cardioembolism was proposed [74–76]. In a consensus paper from 2016, atrial cardiomyopathy was defined as “any complex of structural, architectural, contractile or electrophysiological changes affecting the atria with the potential to produce clinically-relevant manifestations,” including AF and stroke [77]. Underlying structural changes of the atria might be visualized using TTE/TEE, CT, or MRI [77, 78]. It remains to be established whether atrial cardiomyopathy and its proposed biomarkers alone are sufficient for thrombus formation with subsequent stroke or merely serve as surrogate parameters for subclinical AF [79].

Cardiac imaging provides valuable information for further management of patients with unknown stroke etiology. Guidance on who to screen for AF is urgently needed considering that recent studies in ESUS patients failed to demonstrate a beneficial effect of empirical anticoagulation [80, 81]. Furthermore, despite documented overall cost-effectiveness, prolonged ECG monitoring is often restricted due to higher cost at the individual level and limited man power as well as technical resources [11, 82]. Beside patients’ characteristics such as older age, laboratory, as well as ECG findings, echocardiography might aid patient selection for prolonged ECG monitoring [11, 13, 83, 84]. Echocardiography, in particular TTE, seems to be a reasonable method to guide AF screening considering its comparatively low costs and wide availability [85]. Left atrial volume, preferably indexed on body surface area (LA volume index), has been demonstrated to be predictive for the detection of incident AF [85–87]. Quantification of LA volume can be further optimized using three-dimensional TTE [88]. Furthermore, parameters of LV diastolic dysfunction and atrial function might serve as indicators for an increased risk of incident AF [89, 90]. In a recent retrospective single-center study on 531 cryptogenic stroke patients, low atrial strain as well as global longitudinal strain of the LV showed incremental value for AF risk prediction over LA volume, LV ejection fraction and E/e’ ratio [91•]. This is in line with results from another retrospective single-center study on 616 cryptogenic stroke patients [92]. In a recent small prospective single-center study with 56 cryptogenic stroke patients who had a cardiac monitor implanted for AF monitoring, low left atrial strain predicted incident AF independent of classical parameters of diastolic dysfunction, global longitudinal LV strain and cardiovascular risk factors [93]. Low atrial strain has further been shown to correspond with atrial fibrosis detected on cardiac MRI and might therefore be a good surrogate parameter for atrial remodeling [94, 95]. However, these new parameters are based on speckle tracking imaging and might not yet be routinely assessed.

The time interval from the beginning of the P wave on the surface ECG to the peak of the A′ wave obtained by tissue Doppler imaging (TDI), the so-called PA-TDI, is an easily derivable echocardiographic parameter that reflects total atrial conduction time. It is inversely correlated with LA-strain and is associated with atrial fibrosis as well [96, 97]. An increased PA-TDI has been shown to be predictive of incident AF in different patient cohorts, including patients with cryptogenic stroke [98–100]. While the detection of spontaneous echo contrast or even thrombi in the LAA is associated with increased risk for mortality and LAA morphology has been linked to thromboembolic risk, it is uncertain whether they are relevant predictors of AF detection after a stroke [101–103]. Valvular abnormalities (particularly advanced atrioventricular valve diseases) are also associated with AF in stroke patients [83].

## Guideline Recommendations

The European Stroke Organization guideline issued in 2008 recommends echocardiography in selected ischemic stroke or TIA patients, e.g., “in suspected cardiac source of embolism,” “evidence of cardiac disease on history, examination, or ECG” or if “no other identifiable causes of stroke” is identified (class III, level B recommendation) [27]. However, no recommendation is given as to the choice of TTE or TEE. In a recent consensus statement from the European Stroke Organization-Karolinska Stroke Update Conference, TTE was considered the primary choice for cardiac imaging (grade A), while TEE and bubble test-transcranial Doppler were recommended in patients with ESUS for detection of a PFO (grade A) as well as TEE over TTE to detect aortic atheroma (grade C) [84]. The recently updated American Heart Association guideline on acute stroke management considers echocardiography reasonable in selected ischemic stroke patients to guide secondary stroke prevention (class IIa recommendation, expert opinion) and to determine whether eligibility criteria for a PFO closure are met in patients with ESUS (class IIa recommendation, evidence from randomized trials) [104]. The American Society of Echocardiography guidelines were published in 2016 and recommend routine use of TTE as a screening tool for potential cardiac sources of embolism in case of an ischemic stroke or a TIA, while TEE might be considered as an initial or supplemental test in specific cases, e.g., suspicion for endocarditis [105]. Cardiac CT and MRI should be reserved to selected patients with high suspicion for cardioembolism and inconclusive results after echocardiography [105]. Due to its semi-invasive nature, TEE is not recommended if potential results will not change therapeutic decisions [105]. The European Association of Echocardiography recommendations, published in 2010, consider TTE the primary choice for cardiac imaging after ischemic stroke in most individuals. However, TEE is additionally recommended in specific cases despite negative finding on TTE, e.g., in case of suspicion for endocarditis, a PFO or for the evaluation of prosthetic valves [3]. The Canadian Stroke Best Practice Recommendations for Acute Stroke Management state that echocardiography should be considered in patients with suspected embolic stroke or TIA and normal neurovascular imaging without a contraindication for OAC [106].

## Conclusions

Echocardiography constitutes the primary choice for cardiac imaging after acute ischemic stroke with TTE and TEE providing complementary information (Table 2). TTE accurately visualizes cardiac function and should be the first choice in most patients considering its non-invasive character and wide availability. TTE findings might also help to select patients undergoing prolonged rhythm monitoring for a first episode of AF. TEE can provide additional information, e.g., on left atrial pathologies, and should in particular be used in patients with suspected cardioembolic stroke. Cardiac CT or MRI are a valuable alternative in specific situations but cannot be recommended as primary imaging methods at the moment. Further imaging studies in patients with acute ischemic stroke or TIA are needed to better guide clinical practice. Of note, the prognostic benefit of cardiac imaging guided therapy changes following ischemic stroke or TIA has yet to be demonstrated.

**Table 2:** Proposed diagnostic approach to cardiac imaging in patients with ischemic stroke

Medical history, patient characteristics, laboratory, ultrasound, ECG and brain imaging findings			
Presumed stroke etiology	AF	Determined etiology (exceptcardioembolism) at cardiovascular disease risk	Suspected (cardio-) embolism/unknown
Recommended imaging method	TTE	TTE	TTE and TEE
Alternative imaging method	TEE (if presence of left atrial thrombus impacts on timing of OAC)	**–**	Cardiac CT/MRI in case of contraindications for TEE/inconclusive results
Timing of imaging	During in-hospital stay	During in-hospital stay or after discharge	During in-hospital stay
Imaging objectives	• Screening for concomitant structural abnormalities/(coronary) heart disease • Stroke risk assessment • Guiding timing of starting / continuing OAC	• Screening for competing stroke mechanisms • Screening for concomitant structural abnormalities/(coronary) heart disease	• Screening for potential source of cardioembolism • Screening for concomitant structural abnormalities/(coronary) heart disease • Prediction of incident AF during long-term ECG monitoring

*AF* atrial fibrillation, *CT* computed tomography, *ECG* electrocardiogram, *TEE* transoesophageal echocardiography, *TTE* transthoracic echocardiography, *MRI* magnetic resonance imaging, *OAC* oral anticoagulation

## Abbreviations

AF: Atrial fibrillation
ASA: Atrial septum aneurysm
CAD: Coronary artery disease
CT: Computed tomography
ECG: Electrocardiogram
ESUS: Embolic stroke of undetermined source
LA: Left atrium/atrial
LAA: Left atrial appendage
LV: Left ventricle/ventricular
MI: Myocardial infarction
MRI: Magnetic resonance imaging
OAC: Oral anticoagulation
PFO: Patent foramen ovale
TEE: Transesophageal echocardiography
TIA: Transient ischemic attack
TTE: Transthoracic echocardiography
